# Nanoindentation Creep Behavior of Additively Manufactured H13 Steel by Utilizing Selective Laser Melting Technology

**DOI:** 10.3390/ma17153756

**Published:** 2024-07-30

**Authors:** Evangelos Giarmas, Emmanouil K. Tzimtzimis, Nikolaos Kladovasilakis, Dimitrios Tzovaras, Dimitrios Tzetzis

**Affiliations:** 1 Digital Manufacturing and Materials Characterization Laboratory, School of Science and Technology, International Hellenic University, 14th km Thessaloniki-Moudania, Thermi, 57001 Thessaloniki, Greece; e.giarmas@ihu.edu.gr (E.G.);; 2 Centre for Research and Technology Hellas—Information Technologies Institute (CERTH/ITI), 57001 Thessaloniki, Greece

**Keywords:** additive manufacturing, selective laser melting, creep properties, nanoindentation

## Abstract

Nowadays, H13 hot work steel is a commonly used hot work die material in the industry; however, its creep behavior for additively manufactured H13 steel parts has not been widely investigated. This research paper examines the impact of volumetric energy density (VED), a critical parameter in additive manufacturing (AM), and the effect of post heat-treatment nitrification on the creep behavior of H13 hot work tool steel, which is constructed through selective laser melting (SLM), which is a powder bed fusion process according to ISO/ASTM 52900:2021. The study utilizes nanoindentation tests to investigate the creep response and the associated parameters such as the steady-state creep strain rate. Measurements and observations taken during the holding phase offer a valuable understanding of the behavior of the studied material. The findings of this study highlight a substantial influence of both VED and nitrification on several factors including hardness, modulus of elasticity, indentation depth, and creep displacement. Interestingly, the creep strain rate appears to be largely unaltered by these parameters. The study concludes with the observation that the creep stress exponent (*n*) shows a decreasing trend with an increase in VED and the application of nitrification treatment.

## 1. Introduction

Additive manufacturing (AM) has become a major focus of research in recent years. This process involves building by precisely adding material layer by layer. AM offers advantages over traditional manufacturing, such as faster production, reduced waste, superior customization, and the ability to create near-net shapes. Laser-based AM is particularly useful for metallic parts in various industries. However, AM’s inherent complexity, arising from multiple design and process variables, poses challenges for researchers and limits the availability of commercial technologies [1,2,3].

Driven by the rising demand for AM, particularly selective laser melting (SLM), the need for improved process development, especially for novel powder materials, has intensified. In SLM, a high-power laser melts a layer of powder, building a solid part layer by layer. The laser power, layer thickness, hatch width, and scanning speed are crucial parameters that control the build process and significantly impact both the build rate and the mechanical properties of the final part. Each material requires a specific set of these parameters to achieve desired qualities like high density and surface roughness [4,5,6]. SLM enables the rapid production of complex, functional metallic parts. Like other AM methods, SLM allows the creation of intricate geometries and features that are challenging or impossible with traditional methods like machining or pressure casting. It also offers the unique ability to manufacture parts with intricate internal cooling channels and lattice structures, and consolidate multiple components into single, functional units [6,7,8,9,10,11,12,13]. This versatility, coupled with the wide availability of various metallic powders, makes SLM a highly attractive AM technology.

To ensure that metal AM parts are suitable for industrial use, it is important to understand their mechanical behavior. This necessitates conducting experiments to assess vital mechanical behavior such as tensile strength and elongation. Numerous studies have already explored the impact of various printing configurations (like process parameters, build orientation, and so on) on the mechanical performance of H13 tool steel components produced through additive manufacturing [14,15,16,17,18,19]. However, beyond tensile testing, nanoindentation is a commonly employed technique for assessing mechanical properties at submicron levels and holds significant value for experimental studies in the basic physics of materials. It facilitates the identification of individual occurrences like the activation of dislocation sources, the onset of shear instability, and phase changes during the testing process, by leveraging high-precision load-displacement data [20].

Recently, there has been a strong focus on studying the creep behavior and mechanisms of micro- and nanostructured materials and thin films using nanoindentation creep tests [21,22]. Nanoindentation creep experiments offer several distinct benefits over conventional uniaxial creep tests. They can precisely measure localized creep characteristics like those of a thin film applied to a substrate, which cannot be determined using traditional uniaxial creep tests. Additionally, nanoindentation creep methods do not require a significant specimen volume, and multiple repeated tests can be conducted on a single sample. The duration of these tests is also considerably less than that of a standard uniaxial creep test [23]. Nguyen et al. [24] investigated the nanomechanical properties of H13 steel fabricated using the SLM method. The study focused on the interrelationship between the nanoindentation strain rate and hardness. The strain-rate sensitivity exponent (m = 0.022) of this material indicated that the nanoindentation hardness increased in a range of 8.41–9.18 GPa with an increase in the strain rate ranging from 0.002 to 0.1 s^−1^. In another research, the main focus was the relationship between the nanoindentation strain rate and hardness. The strain-rate sensitivity values were 0.022, 0.019, 0.027, 0.028, and 0.035 for SLM H13 at laser scan speeds of 100, 200, 400, 800, and 1600 mm/s, respectively. This indicates that the hardness increases as the strain rate increases. Notably, the hardness values of the SLM H13 at the 200 mm/s laser scan speed are the highest and least sensitive to the strain rate compared to the H13 samples at other scan speeds [25,26].

**Figure 1:**
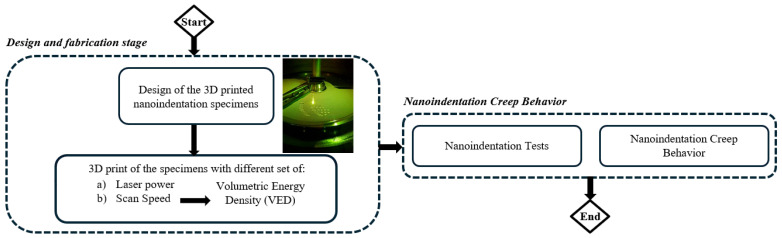
Flowchart of the current research work.

In the current paper, the influence of crucial process-related additive manufacturing parameters on the mechanical and creep behavior of H13 hot work tool steel was investigated with the aid of nanoindentation tests. Additionally, the influence of nitrification has been studied. More specifically, the influence of volumetric energy density (VED) on the creep displacement, the creep strain rate, and the creep stress exponent (*n*) was studied. Other similar studies have utilized a generalized physical model (so-called “Maxwell–Voigt”) in order to reveal the plastic origin of high-entropy metallic glasses [27]. The presented research is very critical, because not many similar studies are available in the literature concerning AM H13 hot work tool steel. The primary objective of this study is the investigation of the creep behavior of additively manufactured H13 hot work tool steel, with the intent of integrating it into standard industrial operations as a substitute for traditionally produced steel. Given the lack of comparable research, this investigation is poised to yield numerous significant findings. Figure 1 depicts the applied methodology of the present research paper.

## 2. Material and Methods

### 2.1. Materials and Sample Preparation

The material that has been selected to be investigated is the OERLIKON MetcoAdd™ H13-A (Westbury, NY, USA), an air-hardenable, iron-chromium martensitic steel powder. Its chemical composition according to the manufacturer is shown in Table 1.

To achieve the desired mechanical behavior of H13 hot work tool steel, various printing parameters were considered. The energy imparted to the material is a very important variable in the SLM process, because it directly affects the characteristics of the molten pool during formation and, consequently, the properties of the final part. This energy is known as volumetric energy density (VED), measured in J/mm^3^, and is described as follows:

(1) $$\mathrm{VED}=\frac{P}{V*h*t}\left[\frac{J}{{\mathrm{mm}}^{3}}\right]\;\;$$

where P is the laser power (J/s), V is the scanning speed (mm/s), h is the hatch width (mm), and t is the layer thickness (mm). Katancik et al. explored a range of printing parameters [28]. The component with the highest density, exhibiting a relevant density of 99% and a porosity of less than 0.01%, was fabricated utilizing a volumetric energy density of 760 J/mm^3^. This was accomplished with settings of 152 W laser power, 100 mm/s scanning speed, 40 mm hatch spacing, and 50 mm layer thickness. In the current work, the hatch width was selected constant at 40 μm, and the layer thickness at 25 μm. The variables were the scanning speed and the laser power. The different combinations of printing parameters are presented in Table 2.

### 2.2. Nanoindentation Test

Nanoindentation experiments were conducted using a Shimadzu DUH-211S instrument (Kyoto, Japan) equipped with a Berkovich diamond tip (included angle: 65°, tip radius: 100 nm). Cylindrical specimens were used for the conducted nanoindentation tests. The calculation method of Oliver and Pharr has been used in order to determine the indentation hardness and the elastic modulus of the SLM printed parts [29]. The indentation’s maximum depth in the following function can be used in order to determine the hardness (H):

(2) $$H=\frac{P_{max}}{A}$$

where P_max_ is the maximum applied load measured at the maximum depth of penetration (h_max_), and A is the projected contact area between the indenter and the film. For a perfect Berkovich indenter, A can be calculated as a function of the contact indentation depth h_f_ as

(3) $$A=33\sqrt{3}h_{f}^{2}\tan^{2}65=23.96h_{f}^{2}$$

The contact indentation, known as *h_f_*, can be calculated using the following formula:

(4) $$h_{f}=h_{max}-\varepsilon \frac{P_{\max}}{s}$$

where *ε* is a geometric constant that takes a value of 0.75 for an indenter with the shape of a pyramid, and *S* is the contact stiffness. The contact stiffness, *S*, can be revealed as the gradient of the unloading curve at the point of maximum load as

(5) $$s={\left(\frac{\mathrm{dP}}{\mathrm{dh}}\right)}_{h=h_{\max}}$$

The reduced elastic modulus *E_r_* is given by

(6) $$E_{r}=\frac{S}{2\beta }\sqrt{\frac{\pi }{A}}$$

where *β* is a constant that is determined by the shape of the indenter. For the Berkovich indenter used in this case, the value of *β* is set to 1.034. Following this, the elastic modulus of the specimen (*E_S_*) can be computed as per the following formula:

(7) $$\frac{1}{E_{r}}=\frac{1-v_{s}^{2}}{E_{s}}+\frac{1-v_{i}^{2}}{E_{i}}$$

where *v_i,s_* and *E_i,s_* describe the Poisson’s ratio and elastic modulus, respectively. Furthermore, for a diamond indenter, the Poisson’s ratio (*v_i_*) is 0.07 and the elastic modulus (*E_i_*) is 1140 GPa. The specimen’s elastic modulus (*E_S_*) and hardness (*H*) are calculated using the equations mentioned above.

The instrument possesses a load resolution of 0.196 μN. All tests were performed at room temperature. The nanoindentation procedure involved applying a controlled load profile to the surface of the filaments via the diamond tip. The load profile consisted of a continuous increase to a peak value of 800 mN, held constant for a dwell time of 3 s (creep time), followed by a complete unload to zero force. The indentation depth was continuously monitored as a function of the applied load. The average of 10 individual measurements was employed to determine the elastic modulus and hardness of the material.

### 2.3. Nitrification

**Figure 2:**
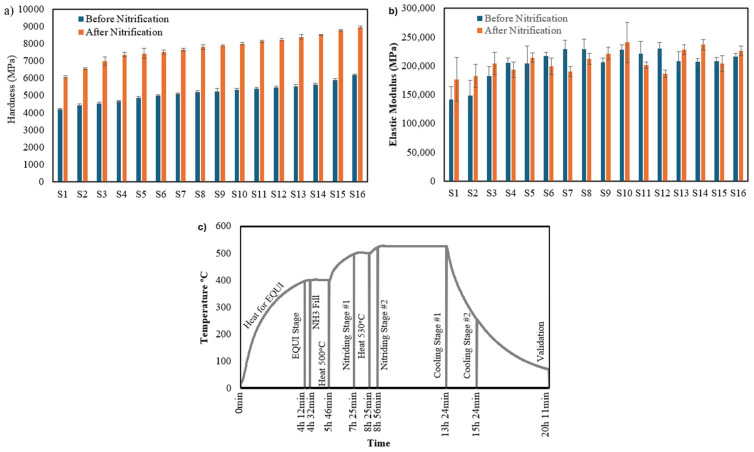
(**a**) Indentation hardness of H13 hot work tool steel, (**b**) modulus of elasticity before and after the nitrification process, and (**c**) nitrification temperature–time program.

Concerning the next step of the nanoindentation experiments, the specimens underwent surface treatment via nitridation to investigate their influence on mechanical behavior. The nitrification program employed a two-stage cooling process and it is depicted in Figure 2. The program encompassed a total duration of 20 h and 11 min. During the initial phase (4 h and 12 min), the temperature steadily increased to reach the target value of 397 °C. Subsequently, ammonia gas was introduced into the furnace chamber (4 h and 42 min). The temperature was then elevated further to 500 °C within the next hour (5 h and 46 min). This stage marked the initiation of the first nitriding step, which concluded at 8 h and 25 min. The second stage commenced with another temperature ramp, reaching a peak of 530 °C at 13 h and 24 min. This stage concluded the nitriding process. A two-step cooling sequence followed. The first stage reduced the temperature to 255 °C by 15 h and 24 min. The final cooling stage brought the temperature down to 70 °C, signifying the completion of the nitridation program at 20 h and 11 min.

## 3. Results and Discussion

### 3.1. Nanoindentation Test Results

Figure 2 depicts the results for the hardness and the modulus of elasticity. For all the selected parameters, 10 sets of specimens were fabricated for each printing parameter and the standard deviation of them is also depicted in this figure. Specimen 16 demonstrated the utmost hardness, reaching 6191 ± 60 MPa. It becomes clear that as the VED increases, there is a corresponding rise in hardness. The greatest modulus of elasticity was observed in specimens 6 through 14 and 16, each surpassing 215,000 MPa. On the other hand, specimens 1 and 2 presented the minimal elastic modulus, falling below 150,000 MPa. These reduced modulus values are due to the use of varied printing parameters, which were inadequate for achieving the required mechanical performance standards. In terms of indentation depth, specimen 16 showed the most advantageous properties, with an indentation depth of 2.11 ± 0.01 μm. The largest indentation depth was recorded for specimen 1 at 2.7 ± 0.01 μm, a result of choosing additive manufacturing parameters that did not meet the necessary mechanical performance benchmarks.

Nitrification significantly enhanced the nanoindentation behavior of H13 hot work tool steel, making it more resistant to the extreme conditions of extrusion. Hardness increased by up to 51.23% (Table 3), with specimen 16 exhibiting the highest value (8960 ± 77 MPa) after treatment. The effect on elastic modulus was less pronounced, with an average increase of around 4.3%. However, all specimens displayed a modulus exceeding 215,000 MPa.

### 3.2. Microstructural Characterization

**Figure 3:**
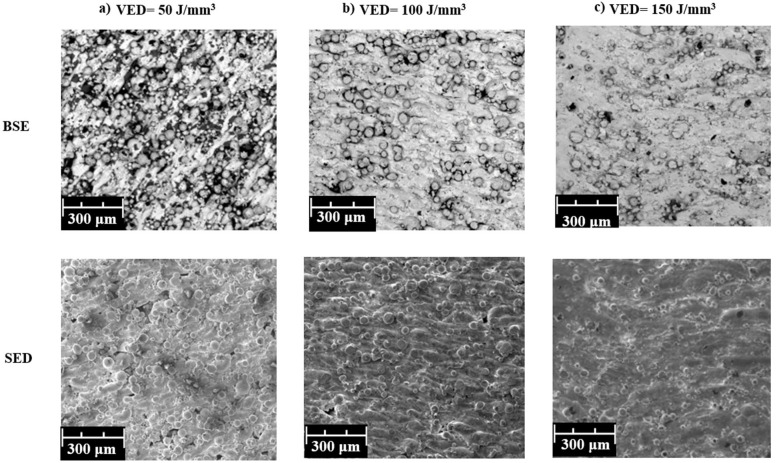
SEM images of the specimens: (**a**) low energy density (50 J/mm^3^), (**b**) medium energy density (100 J/mm^3^), (**c**) high energy density (150 J/mm^3^).

The microstructure of the additively manufactured metal parts was investigated using a Phenom ProX instrument (ThermoFisher Scientific, Waltham, MA, USA) scanning electron microscope (SEM) equipped with both a backscattered electron detector (BSD) and a secondary electron detector (SED) for enhanced characterization. This combination of detectors ensured a comprehensive analysis of the microstructure. Figure 3a illustrates the microstructure of specimens fabricated with a low energy density. Large areas of unmelted powder particles are evident, indicating incomplete fusion with the surrounding material. These unfused regions contribute to surface irregularities and microstructural discontinuities, which could potentially have a detrimental effect on the mechanical performance of the final parts. To illustrate this point further, Figure 3a presents higher magnification SEM images of specimens produced with a low energy density (50 J/mm^3^). Unmelted particles are clearly visible within the microstructure. Conversely, as the energy density during printing increased, the presence of these unfused regions diminished. Figure 3b,c, corresponding to energy densities of 100 J/mm^3^ and 150 J/mm^3^, respectively, demonstrate a significant reduction in unmelted particles. This improved melting efficiency translates to smoother surfaces and potentially enhanced mechanical properties.

### 3.3. Nanoindentation Creep Behavior

In order to study the creep behavior with nanoindentation tests, the data collected during the holding stage of the experiment were used. The experimental data during the holding stage could be well-fitted with the following equation [16]:

h = hi + a(t-ti)1/2 + b(t-ti)1/4 + c(t-ti)1/8(8)

where h is the indenter displacement during the holding stage; t is creep time; and hi, ti, a, b, and c are the best-fit parameters that came from Equation (8).

**Figure 4:**
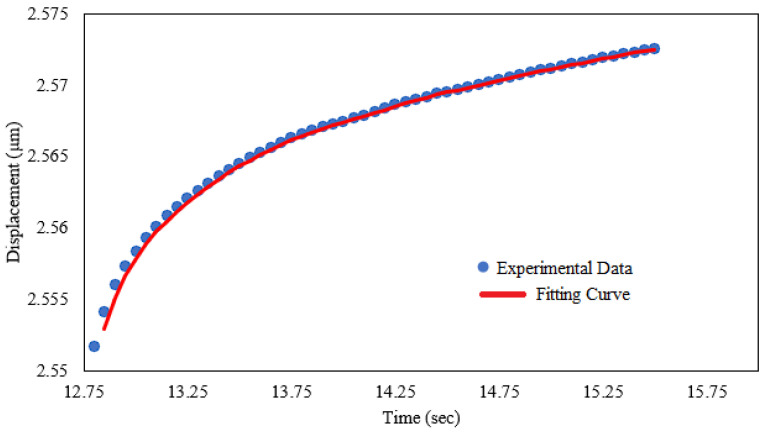
Experimental and fitted creep displacement and time curve for Sample 1 with a VED of 50 J/mm^3^ without nitrification.

The experimental data and fitted creep displacement and time curve for S1 with VED of 50 J/mm^3^ without nitrification during the nanoindentation holding stage are typically depicted in Figure 4. The red line represents the fitted curve according to Equation (8) and the blue dots show the experimental results. Furthermore, Table 4 shows the values of the best-fit parameters from Equation (8). It is clearly revealed that the fitted curve presents an almost perfect agreement with the experimental results for the ambient-temperature creep of the H13 additively manufactured hot work tool steel during the holding stage of the nanoindentation test. The best-fit parameters have been found with the aid of a command line–driven graphing utility (Gnuplot/www.gnuplot.info, accessed from 2 September 2022 until 15 December 2022), where the parameters that satisfy Equation (8) according to the collected data from the experiments have been calculated.

**Figure 5:**
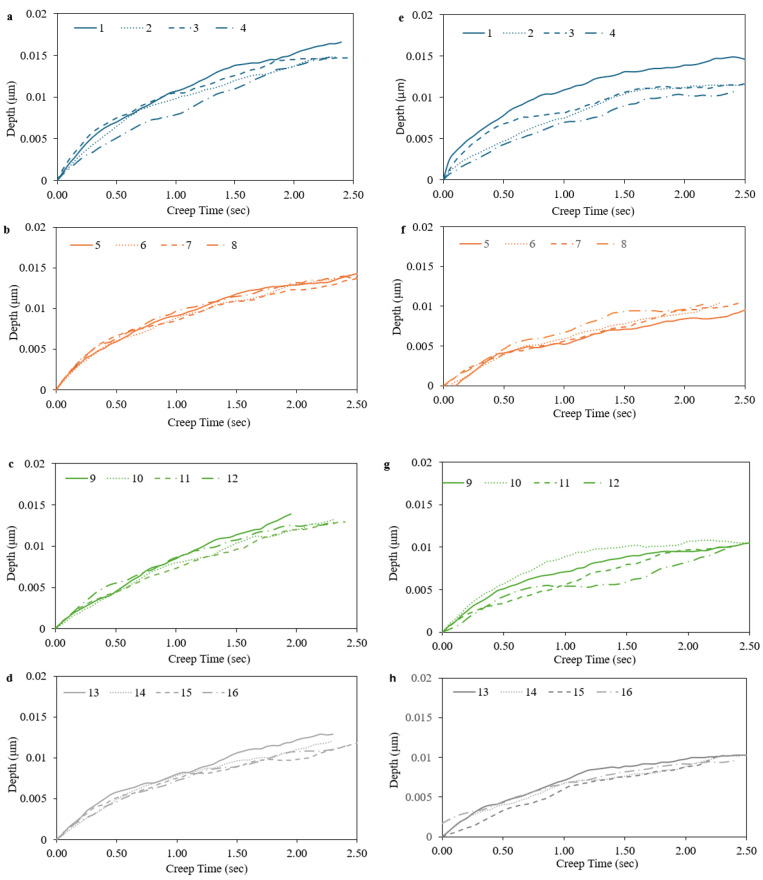
Creep displacement–time curves for different VED conditions without (**a**–**d**)/with (**e**–**h**) nitrification.

The research data presented in Figure 5 illustrate the creep displacement–time curves, which were derived through the process of nanoindentation. These curves represent the outcomes for 16 distinct conditions of volumetric energy density (VED), both with and without the application of a nitrification heat-treating process, as detailed in Table 5. The results of this study underscore the substantial impact of both VED and nitrification on the creep behavior of the material under investigation. In the case of specimens that did not undergo nitriding, an increase in VED from 50 J/mm^3^ to 150 J/mm^3^ led to a notable decrease of 31.32% in creep displacement, changing from 0.0166 μm to 0.0114 μm. Similarly, specimens that were subjected to nitriding displayed a 33.79% reduction in creep displacement, decreasing from 0.0145 μm to 0.0096 μm, when the same VED values were applied. It is worth noting that the process of nitrification consistently resulted in a reduction of creep displacement across all VED conditions, with an average decrease observed to be 24.54%.

These findings highlight the synergistic effect of VED and nitrification on enhancing the creep resistance of H13 hot work tool steel, which was fabricated using the selective laser melting (SLM) technique. The combination of a VED of 150 J/mm^3^ and the application of a nitrification treatment resulted in the lowest observed creep displacement, indicating the potential for optimizing the material’s performance in this regard.

According to Mayo et al. [19], the creep strain rate $\dot{\varepsilon }$ was calculated from displacement through the following equation:

(9) $$\dot{\varepsilon }=\frac{\dot{h}}{h}\;=\frac{1}{h}\frac{dh}{dt}$$

**Figure 6:**
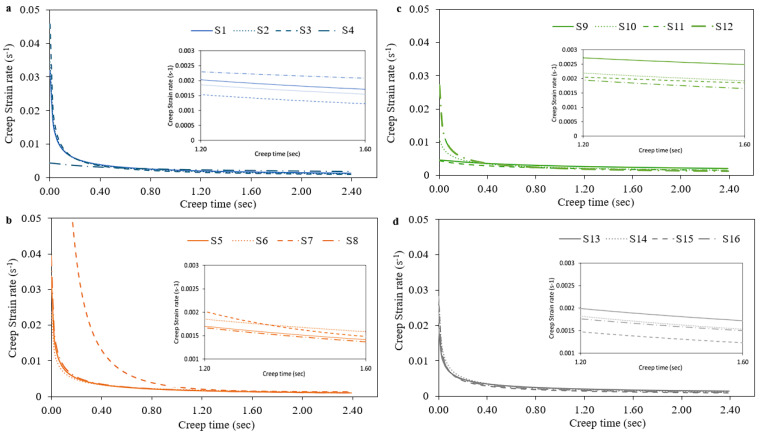
Creep strain–time curves for different VED conditions without nitrification (**a**) S1–S4, (**b**) S5–S8, (**c**) S9–S12 and (**d**) S13–S16.

**Figure 7:**
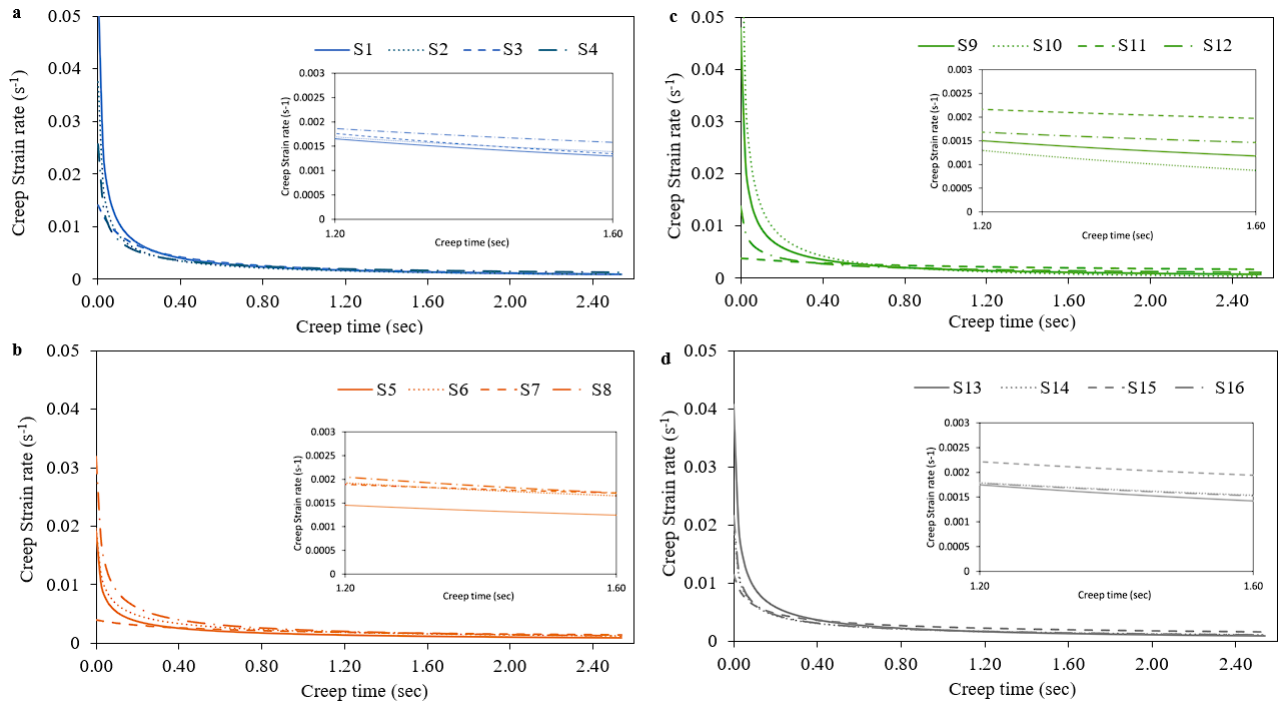
Creep strain–time curves for different VED conditions with nitrification (**a**) S1–S4, (**b**) S5–S8, (**c**) S9–S12 and (**d**) S13–S16.

The creep strain rate data were calculated by differentiating the fitted creep h-t curves in the hold stage with Equation (8) while Figure 6 and Figure 7 depict the creep strain-time curves for the 16 selected volumetric energy density (VED) conditions (S1–S16), both with and without the nitrification heat-treating process. It can be seen that the creep $\dot{\varepsilon }$-t curves consist of the following two stages: a transient one and a steady-state one. At the beginning of the load holding, a very high creep strain rate was measured and then the creep strain rate decreased rapidly. This phenomenon may occur due to work hardening caused by the plastic deformation, corresponding to the transient creep. After a holding time of 2.3 s, an almost steady-state creep phase is depicted and a very slow decrease in the creep strain rate with increasing creep time is revealed [20]. From Figure 6 and Figure 7, it is evident that there is no important change concerning the creep strain rate over the creep time for both VED and nitrification, but a small decrease in the creep strain rate with the use of nitrifications can be detected. Liu et al. concluded to the same insensitivity concerning the steady creep strain rate due to the dislocation creep phenomenon [30]. In each diagram, a detailed image has been superimposed, where the difference between the different aging conditions is more clearly visible.

The dominant creep mechanism and the ambient-temperature creep behavior of the materials can be studied by Equation (10) of the stress (s) and the creep strain rate ($\dot{\varepsilon }$) [21]:

(10) $$\dot{\varepsilon }=\alpha \sigma^{n}\;\;\;\;\;\;\;$$

where *n* is the creep stress exponent and *α* is a material constant. The stress in a nanoindentation test should be connected to the pressure applied by the indenter. From the relationship of H = P/A = P/24.56h_c_^2^ (where P is the holding load and A is the projected contact area of the Berkovich tip), the following equation for the creep stress in the holding stage can be easily assumed based on σ = (H/3)/(h_max_/h)^2^ [22]. Moreover, the creep stress exponent *n* is derived by determining the slope of the ln $\dot{\varepsilon }$ versus lnσ plot according to Equation (11):

(11) $$n=\frac{d\left(ln\dot{\varepsilon }\right)}{d\left(ln\sigma \right)}$$

**Figure 8:**
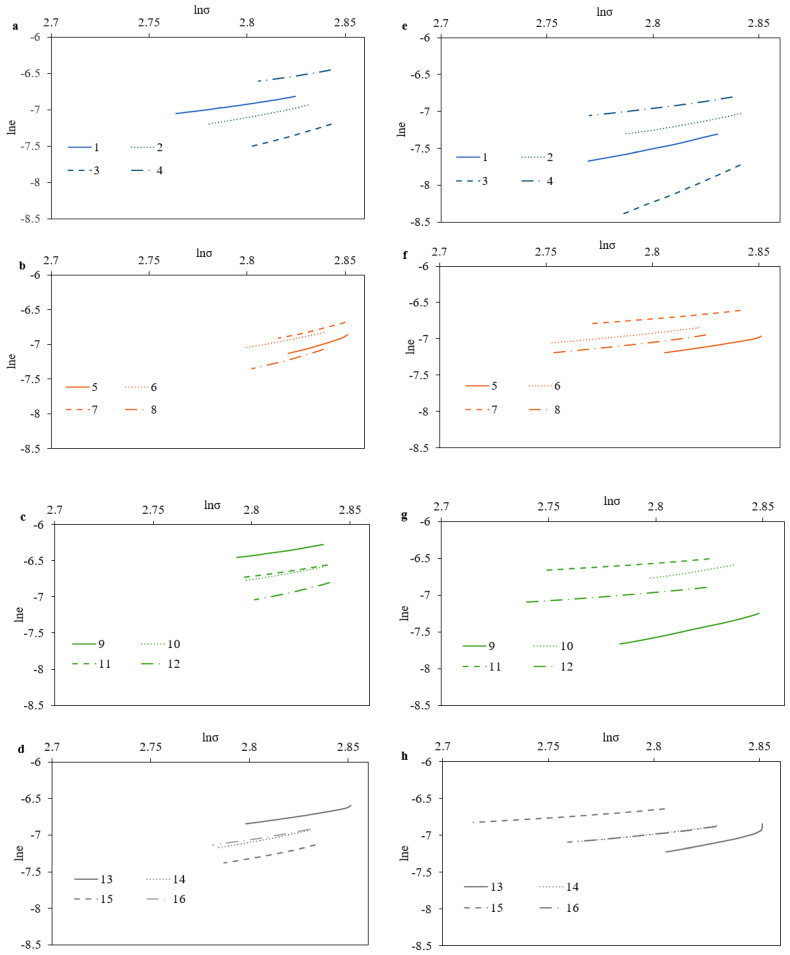
The ln$\dot{\varepsilon }$-lnσ curves for different VED conditions without (**a**–**d**)/with (**e**–**h**) nitrification.

Figure 8 depicts the *ln*$\dot{\varepsilon }$ versus *lnσ* diagrams, using the definitions of creep strain rate and stress, for the 16 selected volumetric energy density (VED) conditions, both with and without the nitrification heat-treating process. In order to make the calculations for each one of the selected specimens according to the different VED conditions and the use of nitrification as a heat-treating process, only the steady-state creep is taken into consideration because the value of the creep stress exponent differs at different locations in the creep strain rate vs. stress curve.

Furthermore, the values of the steady creep strain rate ($\dot{\varepsilon }$) and the creep stress exponent (*n*) for the different VED conditions without and with the stage of nitrification are shown in Table 6 and Table 7, respectively. It is evident that the steady creep strain rate does not vary significantly with the VED values. The values are very low and as a result very sensitive to change that it will not be safe to make a clear assumption. The main reason for this may be that despite the values of VED, there might be important differences to the printing parameters from which the VED are revealed. This might be a good starting point for future research. However, concerning the creep stress exponent (*n*), it seems that with increasing the VED from 50 J/mm^3^ to 150 J/mm^3^, there is a total decrease of 43.32% (from 6.67 to 3.78) for non-nitrided specimens. Similarly, the nitrided specimens exhibited a 50.73% reduction (from 5.48 to 2.70). Finally, nitrification consistently reduced the stress exponent (*n*) across the majority of VED conditions, with an average decrease of 2.94%.

## 4. Conclusions

The effect of volumetric energy density (VED) and the use of nitrification as a heat-treating process on the creep behavior of H13 hot work tool steel produced with the SLM process, and relevant creep parameters such as steady creep strain rate ($\dot{\varepsilon }$) and the creep stress exponent (*n*), were investigated in the presented research using nanoindentation tests. Concerning the hardness, the modulus of elasticity, and the indentation depth, specimen 16 with a VED of 150 J/mm^3^ exhibits the most favorable characteristics both before and after the nitrification process. Nitrification significantly enhanced the nanoindentation behavior of H13 hot work tool steel. Hardness increased by up to 51.23%, with specimen 16 exhibiting the highest value (8960 ± 77 MPa) after heat treatment. The effect on the elastic modulus was less pronounced, with an average increase of around 4.3%. However, all specimens displayed a modulus exceeding 215,000 MPa after nitrification. Moreover, a significant influence of both VED and nitrification on creep behavior has been revealed. Non-nitride specimens displayed a 31.32% reduction in creep displacement (from 0.0166 μm to 0.0114 μm) as the VED increased from 50 J/mm^3^ to 150 J/mm^3^. Similarly, nitride specimens exhibited a decrease in creep displacement of 33.79% (from 0.0145 μm to 0.0096 μm) at these VED levels. Furthermore, nitrification consistently reduced creep displacement across all VED conditions, with an average decrease of 24.54%. Finally, despite that the steady creep strain rate does not vary significantly with the aging process, the creep stress exponent (*n*) seems to decrease by 43.32% (from 6.67 to 3.78) for non-nitrided specimens and 50.73% for the nitride ones.

To sum up, the aforementioned studies pave the path for initiating considerations of the creep behavior in additively manufactured H13 steel components. Given the criticality of creep resistance in the high-performance applications typically associated with these metals, understanding this behavior is paramount.

## Figures and Tables

**Table 1:** PSD, Hall flow, and chemical composition for H13A powder according to the manufacturer.

Nominal Range (μm)		D90 (μm)		D50 (μm)		D10 (μm)		Hall Flow (s/50 g)	
−45 + 15		50		34		21		≤25	
Weight Percent (nominal)									
Fe	Cr		Mo		Si		V		C
Balance	5.2		1.3		1.0		1.0		0.4

**Table 2:** Printing parameters for each set of testing specimens.

Sample	VED (J/mm^3^)	Laser Power (W)	Scanning Speed (mm/s)
S1	50	100	2000
S2	57	100	1750
S3	58	115	2000
S4	66	115	1750
S5	68	135	2000
S6	75	150	2000
S7	77	135	1750
S8	80	100	1250
S9	86	150	1750
S10	92	115	1250
S11	100	100	1000
S12	108	135	1250
S13	115	115	1000
S14	120	150	1250
S15	135	135	1000
S16	150	150	1000

**Table 3:** Comparative nanoindentation results for additively manufactured H13 hot work steel before and after the nitrification process.

VED	Hardness (MPa)		Difference	Elastic Modulus (MPa)		Difference
	Before	After		Before	After	
50	4196.78	6076.76	44.80%	141,520	177,000	25.07%
57	4444.36	6555.67	47.51%	148,280	182,780	23.27%
58	4553.83	6989.61	53.49%	182,900	204,500	11.81%
66	4666.16	7371.27	57.97%	205,100	193,300	−5.75%
68	4880.79	7431.27	52.26%	204,260	214,740	5.13%
75	4992.83	7515.16	50.52%	217,460	199,380	−8.31%
77	5093.64	7658.96	50.36%	229,140	190,700	−16.78%
80	5189.16	7810.58	50.52%	228,860	212,260	−7.25%
86	5235.03	7896.8	50.85%	205,900	221,740	7.69%
92	5334.36	8001.45	50.00%	227,780	240,820	5.72%
100	5386.71	8137.8	51.07%	221,200	201,540	−8.89%
108	5459.57	8232.46	50.79%	230,000	186,680	−18.83%
115	5548.13	8390.42	51.23%	208,280	228,700	9.80%
120	5648.06	8509.95	50.67%	207,360	236,780	14.19%
135	5903.83	8789.17	48.87%	208,440	204,220	−2.02%
150	6191.25	8960.81	44.73%	216,360	226,660	4.76%

**Table 4:** Values of the best-fit parameters and asymptotic Standard Error S1 with a VED of 50 J/mm^3^ without nitrification.

Set of Parameters	Asymptotic Standard Error	
a = 0.00538866	±0.001072	(19.89%)
b = 0.019735	±0.003034	(15.38%)
c = −0.0144336	±0.002013	(13.95%)
hi = 2.55421	±0.0002216	(0.0087%)
ti = 12.6707	±0.01187	(0.09366%)

**Table 5:** Creep displacement for different VED conditions.

Sample Number	VED (J/mm^3^)	Creep Displacement (μm) *	% Change	Creep Displacement (μm) **	% Change
S1	50	0.0166	-	0.0145	-
S2	57	0.0148	−10.84%	0.0119	−17.93%
S3	58	0.0149	0.68%	0.0117	−1.68%
S4	66	0.0148	−0.67%	0.0108	−7.69%
S5	68	0.0143	−3.38%	0.0103	−4.63%
S6	75	0.0142	−0.70%	0.0105	1.94%
S7	77	0.014	−1.41%	0.0104	−0.95%
S8	80	0.0141	0.71%	0.0102	−1.92%
S9	86	0.0139	−1.42%	0.0106	3.92%
S10	92	0.0132	−5.04%	0.0104	−1.89%
S11	100	0.0129	−2.27%	0.0101	−2.88%
S12	108	0.013	0.78%	0.01	−0.99%
S13	115	0.0129	−0.77%	0.0103	3.00%
S14	120	0.0121	−6.20%	0.0101	−1.94%
S15	135	0.0119	−1.65%	0.0102	0.99%
S16	150	0.0114	−4.20%	0.0096	−5.88%

* without nitrification; ** with nitrification.

**Table 6:** Steady creep strain rate ($\dot{\varepsilon }$) for different VED conditions.

Sample Number	VED (J/mm^3^)	Steady Creep Strain Rate (e) *	% Change	Steady Creep Strain Rate (e) **	% Change
S1	50	0.00088045	-	0.000481014	-
S2	57	0.000745593	−15.32%	0.000667245	38.72%
S3	58	0.000542276	−27.27%	0.000226144	−66.11%
S4	66	0.001308202	141.24%	0.000833911	268.75%
S5	68	0.000716387	−45.24%	0.000682166	−18.20%
S6	75	0.000872306	21.76%	0.000856347	25.53%
S7	77	0.000953821	9.34%	0.001076213	25.67%
S8	80	0.000655186	−31.31%	0.000763662	−29.04%
S9	86	0.001507832	130.14%	0.000433518	−43.23%
S10	92	0.001126749	−25.27%	0.001126749	159.91%
S11	100	0.001182228	4.92%	0.001257004	11.56%
S12	108	0.000862596	−27.04%	0.000811422	−35.45%
S13	115	0.000953821	10.58%	0.0006205	−23.53%
S14	120	0.000760173	−20.30%	0.000833777	34.37%
S15	135	0.000634656	−16.51%	0.001090283	30.76%
S16	150	0.000799513	25.98%	0.000830285	−23.85%

* without nitrification; ** with nitrification.

**Table 7:** Creep stress exponent (*n*) for different VED conditions.

VED (J/mm^3^)	Creep Stress Exponent (*n*) *	% Change	Creep Stress Exponent (*n*) **	% Change
50	6.675110931	-	5.486328588	-
57	4.199017749	−37.09%	4.501311944	−17.95%
58	3.229997014	−23.08%	11.39958664	153.25%
66	3.485868021	7.92%	2.971131928	−73.94%
68	5.86734502	68.32%	3.290291092	10.74%
75	4.538255719	−22.65%	2.687519724	−18.32%
77	4.710518564	3.80%	2.031330012	−24.42%
80	6.566776868	39.41%	3.036948594	49.51%
86	2.950171409	−55.07%	5.068478173	66.89%
92	3.842315907	30.24%	4.180542552	−17.52%
100	3.274040411	−14.79%	1.773010121	−57.59%
108	4.855511694	48.30%	1.986774532	12.06%
115	2.568365618	−47.10%	4.159389552	109.35%
120	4.111052477	60.06%	2.877173982	−30.83%
135	4.44471448	8.12%	1.850538219	−35.68%
150	3.780465456	−14.94%	2.704244278	46.13%

* without nitrification; ** with nitrification.

## Data Availability

The raw data supporting the conclusions of this article will be made available by the authors on request.
